# Ascorbic acid exhibits more of a protective effect than estradiol against nephrotoxicity induced by malathion in rats: a histopathological and molecular docking study

**DOI:** 10.55730/1300-0144.5974

**Published:** 2024-10-29

**Authors:** Mohammad ALHILAL, Mahmoud ELSAYED MOHAMED SALEM, Ahmed ALI ALBAKOUSH, Suzan ALHILAL, Basant FARAG, Sobhi M. GOMHA

**Affiliations:** 1 Department of Nursing, Faculty of Health Sciences, Mardin Artuklu University, Mardin, Turkiye; 2 Department of Pathology, Faculty of Medicine, Alasmarya Islamic University, Zliten, Libya; 3 Department of Medical Services and Techniques, Vocational School of Health Services, Mardin Artuklu University, Mardin, Turkiye; 4 Department of Chemistry, Faculty of Science, Zagazig University, Zagazig, Egypt; 5 Department of Chemistry, Faculty of Science, Islamic University of Madinah, Madinah, Saudi Arabia

**Keywords:** Ascorbic acid, estradiol, malathion, necrosis, nephritis, molecular docking

## Abstract

**Background/aim:**

Despite the known harmful effects associated with malathion toxicity in various organs, it continues to be widely used for plant protection and insect control. This study is the first to compare the protective effects of estradiol and ascorbic acid against malathion-induced nephrotoxicity through histopathological assessment and molecular docking analyses.

**Materials and methods:**

This study was conducted using 20 female albino rats that were distributed into sham, malathion, malathion + estradiol, and malathion + ascorbic acid groups. Nephrotoxicity was induced by daily treatment with malathion and the effects of estradiol and ascorbic on nephrotoxicity were evaluated. After 4 weeks of treatment, the animals were sacrificed and the kidneys were examined following hematoxylin and eosin (H&E) staining. Histopathology results were supported by molecular docking studies of estradiol and ascorbic acid against a target protein (PDB ID: 2YMX), the peptide inhibitor Fab408 inhibiting acetylcholinesterase (AChE). The inhibition of AChE is the primary mechanism of the toxic effects of malathion.

**Results:**

Histopathological examination revealed a notable elevation (p < 0.001) in degeneration and necrosis within the tubular epithelium and interstitial nephritis in the malathion group compared to the sham group. Daily administration of estradiol and ascorbic acid resulted in a notable reduction (p = 0.0022) in the severity of these histopathological changes in the malathion + estradiol and malathion + ascorbic acid groups compared to the malathion group. Of these, the most significant decreases were observed in the malathion + ascorbic acid group. Docking studies of these compounds against the selected protein (PDB ID: 2YMX) revealed promising binding scores. Ascorbic acid exhibited the highest docking score (−6.44 kcal/mol), indicating a favorable binding interaction with this protein.

**Conclusion:**

Estradiol and ascorbic acid exert protective effects against malathion-induced nephrotoxicity, whereas ascorbic acid showed superior efficacy compared to estradiol. This result was further supported by molecular docking studies.

## 1. Introduction

Insecticides are ubiquitous in agriculture and play an important role in enhancing agricultural production [1,2]. Malathion is a member of the organophosphate group and remains a widely used insecticide in agriculture [3]. However, organophosphates are known for their rapid absorption and lipophilic properties. Thus, they can reach all organs and tissues, including the kidneys, which can result in significant damage [4]. Malathion is also used to eliminate household insects and pathogenic arthropods and to preserve stored grains [5]. It is readily absorbed through mucous membranes or skin [6] and can reach various organs, such as the kidneys [7]. In rats, malathion is nephrotoxic and affects both the function and structure of the kidney. This leads to various kidney diseases, which are characterized by high morbidity, particularly in critically ill patients [8]. Malathion causes glomerular hypertrophy, possibly due to vascular congestion and bleeding between the renal tubules. In addition, the renal tubules display prominent features of severe degeneration [9]. Mechanisms of malathion toxicity include inhibition of acetylcholinesterase (AChE) [10] and marked production of reactive oxygen species [11] exceeding the ability of cells to manage them, which results in tissue damage. Malathion also decreases the activity of antioxidant enzyme systems, which results in damage to all organs, including the kidneys [5]. Malathion may cause DNA damage [12]; thus, it has been associated with chromosomal abnormalities [13]. Therefore, malathion toxicity in individuals exposed to insecticides, particularly farmers, may be the result of DNA damage and oxidative stress [14]. Estradiol is a powerful antioxidant and the stimulation of estrogenic receptors may prevent kidney damage and diseases caused by oxidative stress, thus protecting tissues from inflammation and fibrosis [15]. Oxidative stress is significantly increased in various organs such as the kidneys after oophorectomy and reduces estrogen levels. This is accompanied by an acceleration of the aging process. In contrast, the administration of estrogen to old ovariectomized mice slowed the aging process and decreased the effects of oxidative stress in various organs [16]. Thus, oxidative stress induced by malathion can be ameliorated by the administration of estradiol. Ascorbic acid is a powerful antioxidant because of its ability to donate electrons. Therefore, it can also protect tissues from oxidative destruction [17]. Ascorbic acid also acts as a strong antiinflammatory compound because of its ability to activate enzymes of the cellular antioxidant system. Ascorbic acid removes free radicals and protects all cellular components from oxidative damage [18]. In a previous study, the protective effects of both estradiol and ascorbic acid against malathion-induced lung damage were demonstrated [19]. Based on this evidence, estradiol and ascorbic acid may attenuate nephrotoxicity induced by malathion. In the present study, we provide insight into the histopathological changes in renal tissues resulting from malathion application and the modulatory role of the administration of estradiol and ascorbic acid on these changes. This study aimed to compare the protective effects of estradiol and ascorbic acid against nephrotoxicity. In addition, the bond energy indicated the potential for bond formation between two structures [20]. Therefore, a molecular docking study was conducted for these molecules against the target enzyme (PDB ID: 2YMX). PDB ID: 2YMX is the code assigned by the RCSB Data Bank based on the 1.9-Ǻ resolution structure coordinates of the peptide inhibitor Fab408 directed against AChE. This peptide inhibits AChE by targeting the backdoor region. AChE terminates cholinergic neurotransmission at neuronal and neuromuscular synapses through hydrolysis of the neurotransmitter acetylcholine [21].

## 2. Materials and methods

### 2.1. Chemicals

**Figure 1:**
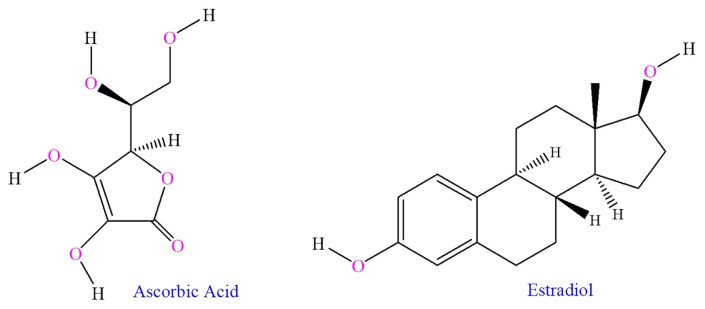
Chemical structures of ascorbic acid and estradiol.

Malathion (99.9% purity), estradiol, and ascorbic acid were obtained from Biogenic (Decatur, IL, USA), NexGen Pharmaceuticals (Mumbai, India), and Almarkazia (Al-Khums, Libya) respectively. The chemical structures of these compounds are shown in Figure 1.

### 2.2. Animals and experiment details

The experimental protocol and animal care followed the ethical standards of the Faculty of Pharmacy, Alasmarya Islamic University, Libya. Ethical approval for the study was obtained under code number AIUS-2020-02.

**Figure 2:**
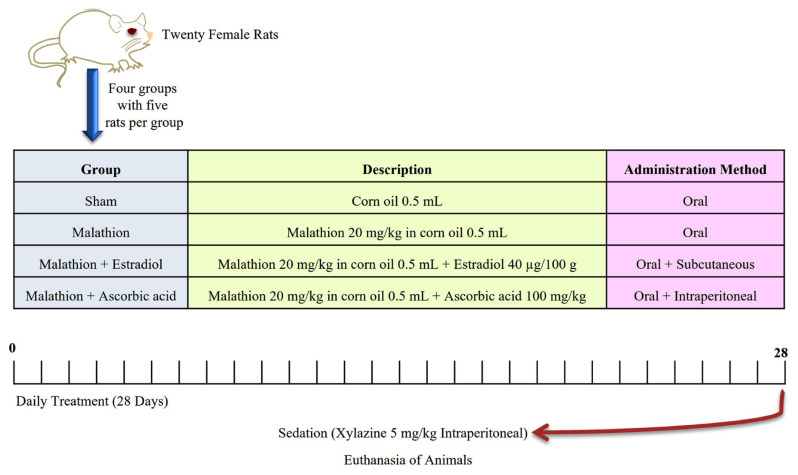
Details of the experimental groups and treatments.

The experiment included 20 female white rats, weighing between 250 and 300 g at approximately 10 weeks of age. Statistical tests related to sample size were conducted using the G*Power program (Version 3.1.9.7, Heinrich Heine University Düsseldorf, North Rhine-Westphalia, Germany), which yielded the following parameters: total sample size = 20, actual power = 0.950, effect size f = 1.0523783, α err. prob. = 0.05, power (1 − β err. prob.) = 0.95, number of groups = 4. This experiment was conducted in the Faculty of Technology of Misurata University, Libya. The rats were housed under standard laboratory conditions with a constant temperature of 25 ± 2 °C and relative humidity of 55 ± 5%. They were housed in polypropylene cages and provided free access to a standard laboratory diet and water ad libitum. The rats were allocated into four groups: the sham group, in which each rat received an oral administration of corn oil at a dose of 0.5 mL; the malathion group, which was orally administered a dose of malathion at 20 mg/kg in 0.5 mL of corn oil; the malathion + estradiol group, which was orally administered malathion with corn oil as above and a subcutaneous administration of estradiol at a dose of 40 μg/100 g; and the malathion + ascorbic acid group, which was orally administered malathion with corn oil as above and an intraperitoneal injection of ascorbic acid at a dose of 100 mg/kg [19]. Estradiol was dissolved in 0.3 mL of sesame oil while ascorbic acid was dissolved in water. The experimental details are summarized in Figure 2.

### 2.3. Histopathological assessment of kidney tissue

A histopathological examination was done following conventional procedures [22]. The kidney tissue samples were fixed in 10% buffered formalin solution and then processed in a routine tissue monitoring procedure. Five-micrometer-thick sections from the processed tissues were taken onto slides, stained with hematoxylin and eosin (H&E) dye, and examined under a light microscope (BX51, Olympus, Tokyo, Japan). According to histopathological features, stained sections were evaluated as absent (−), mild (+; 0–5 cells), moderate (++; 5–10 cells), or severe (+++; >10 cells) [23].

### 2.4. Statistical analysis

For histopathological examination, first the Kolmogorov–Smirnov test, a normality distribution test, was applied. The Kruskal-Wallis test, for nonparametric analysis, was utilized to evaluate differences among groups for semiquantitative data. Paired group comparisons were performed using the Mann-Whitney U test. Statistical analyses were conducted using GraphPad Prism 8.0.2 software (GraphPad Inc., La Jolla, CA, USA).

### 2.5. Molecular docking study

For ligand preparation, molecular modeling was conducted using Molecular Operating Environment software (Chemical Computing Group, Montreal, QC, Canada). The substances were initially sketched using ChemDraw 12.0 (Informer Technologies, Los Angeles, CA, USA). All minimizations were executed until a root mean square deviation (RMSD) gradient of 0.1 kcal mol^−1^ Å^−1^ using the Merck molecular force field at 94×.

For protein preparation, the X-ray diffraction structure of the enzyme (PDB ID: 2YMX, resolution: 1.90 Å) was downloaded in PDB format from the Protein Data Bank [21]. For preparation of the enzyme for the docking study, water was removed. Hydrogen atoms were then added to the structure with the usual geometry and the bonds were reconnected to fix the potential. Dummy atoms were added to the enzyme structure using Alpha Site Finder for a large site search in the enzyme structure [24]. The binding pocket of the protein was then marked and the interaction of the ligand with the amino acids in the active site was examined. Docking was performed using the Triangle Matcher placement method and the London dG score tool. Following docking, the 2D and 3D interactions with amino acid residues were visualized. All docking operations and scoring procedures were documented according to established protocols [25–27].

## 3. Results

### 3.1. Histopathological examination

**Figure 3:**
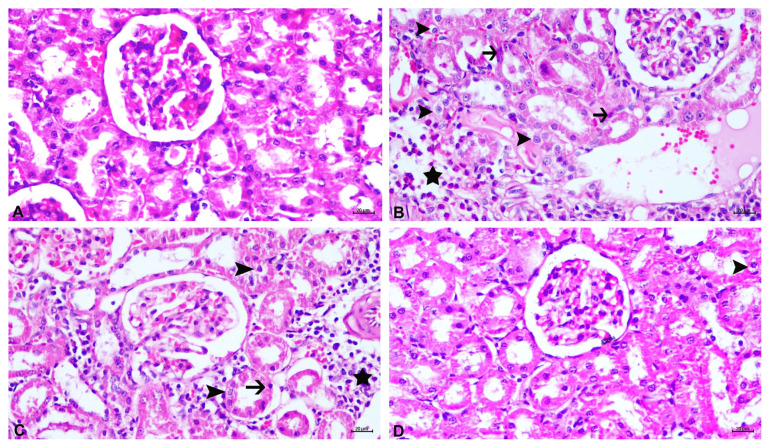
Photomicrography of histological sections of renal tissues of rats. A) Sham group, B) malathion group, C) malathion + estradiol group, D) malathion + ascorbic acid group. Degeneration of tubular epithelium (arrowheads), necrosis (arrows), interstitial nephritis (stars). H&E, bar = 20 μm.

As shown in Figure 3A, the histological examination of sections prepared from the sham group revealed that the cortex and medulla of the kidneys exhibited a normal tissue structure; however, the histological sections obtained from the kidneys of animals in the malathion group revealed notable degeneration and necrosis of the tubular epithelium, along with severe interstitial nephritis, as depicted in Figure 3B.

**Figure 4:**
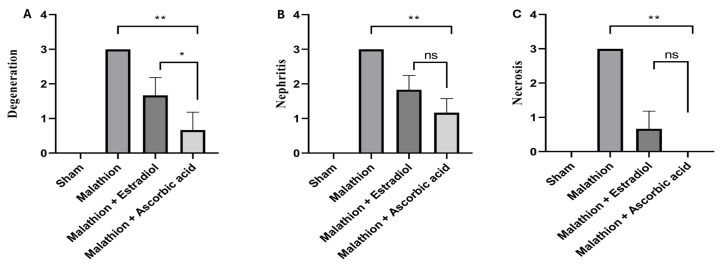
Statistical analysis of histopathological findings in renal tissues of rats. A) Degeneration level significantly decreased in malathion + ascorbic acid group compared to the malathion and malathion + estradiol groups. B) Nephritis level significantly decreased in malathion + ascorbic acid group compared to the malathion group. C) Necrosis level significantly decreased in malathion + ascorbic acid group compared to the malathion group. Results are expressed as mean ± SD. p < 0.05 was accepted to be the lower limit of significance. **: p = 0.0022; *: p = 0.0325; ns: nonsignificant difference (for nephritis p = 0.0801, for necrosis p = 0.0606).

Estradiol application at a dose of 40 μg/100 g body weight to the animals in the malathion + estradiol group and ascorbic acid administration to rats in the malathion + ascorbic acid group at a dose of 100 mg/kg body weight reduced the severity of these changes, as shown in Figures 3C and 3D, respectively. Compared to the malathion + estradiol group, the malathion + ascorbic acid group exhibited a notable decrease (p = 0.0325) in tubular epithelial degeneration, as shown in Figure 4A, although the reductions in nephritis (p = 0.0801) and necrosis (p = 0.0606) were not statistically significant, as shown in Figures 4B and 4C, respectively.

### 3.2. Molecular docking study

**Figure 5:**
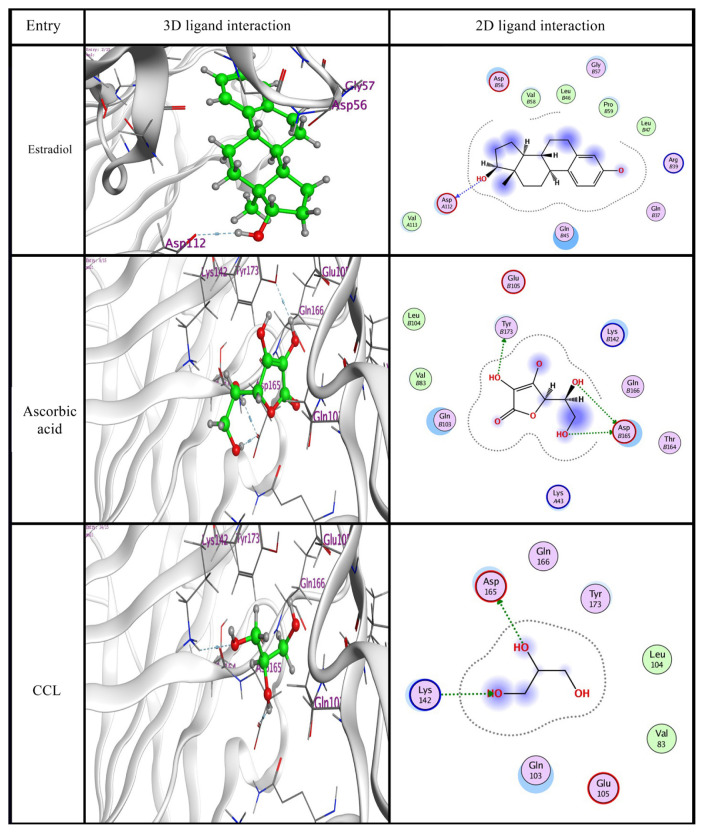
The 3D and 2D ligand interactions within the binding site of 2YMX for designed compounds and CCL.

The interaction of ligands with the active pocket of the target protein was identified using the molecular docking computational technique. Every docking process was based on the following points: (i) The binding energy of our ligands was found to be more negative than that of the cocrystallized ligand (CCL), implying that they fit with higher stability than the CCL. (ii) The interactions seemed to include every potential kind, including H-donor and H-acceptor interactions. (iii) Residues including Asp112, Asp165, Tyr173, and Lys142 were determined to be the most significant residues for key binding receptor backbones. (iv) The oxygen atom sites were the key objectives of the ligation mode. Based on the binding affinity scores and the presence of significant hydrogen bonds, the docked molecules were ranked as shown in Table. According to the simulation, the ascorbic acid ligand had higher binding energy and lower RMSD compared to the estradiol ligand. The results indicated comparable positions and orientations within the ligands to the binding site of the target enzyme. For the estradiol ligand, the oxygen atom interacted as an H-bond donor with Asp112 as shown in Table. A 3D model of the ascorbic acid ligand showed three hydrogen bond donors, two between its oxygen atom with Asp165 and another with Tyr173, as shown in Figure 5. The significance of the hydrogen bonds in determining the specificity of ligand binding has been previously explained [28,29]. Finally, in the cocrystalline ligand, one hydrogen bond donor between its oxygen atom with Asp165 and another H-bond acceptor between its oxygen atom with Lys142 were observed.

## 4. Discussion

We examined the histopathological changes in renal tissues resulting from malathion administration as well as the protective role of estradiol and ascorbic acid against malathion-induced nephrotoxicity. Although many studies have examined malathion-induced organ toxicity, this is the first to compare the protective effects of estradiol and ascorbic acid against malathion-induced nephrotoxicity using histopathological assessment and molecular docking analysis. In the present study, malathion treatment of rats at a dose of 20 mg/kg (1/100 of the oral LD_50_ = 2000 mg/kg body weight) [30] resulted in degeneration and necrosis in the tubular epithelium along with interstitial nephritis. These results are consistent with previous studies indicating that malathion induces significant degeneration in all components of the nephrons [5,31]. The infiltration of inflammatory cells, such as lymphocytes and macrophages, was also observed along with interstitial nephritis [31] and severe hyperemia with necrosis [32].

Malaxon, a metabolite of malathion produced by the cytochrome P450 system, binds to the active site of AChE. This binding action inhibits AChE and the phosphorylation and hydrolysis of acetylcholine. Consequently, this neurotransmitter accumulates, ultimately causing toxicity [33,34]. Oxidative stress is the primary characteristic of malathion poisoning as it induces the production of free radicals and depletes the antioxidant defense system [33]. Organophosphates induce oxidative stress, which interferes with the normal function of tissues and organs, resulting in abnormalities in most organs, including the kidneys [35]. Organophosphate compounds also induce an inflammatory response [33]. These mechanisms explain the nephrotoxicity induced by malathion in female albino rats observed in the present study. In addition, we believe that managing oxidative stress can suppress malathion-induced nephrotoxicity. Degeneration and necrosis of the tubular epithelium and interstitial nephritis were ameliorated following treatment with estradiol and ascorbic acid. These findings are consistent with those of Alhilal and Salem [19], who reported that estradiol and ascorbic acid have protective effects against pulmonary toxicity induced by malathion. Moreover, other experimental studies showed that vitamin C has a protective effect against testicular toxicity [36], hepatotoxicity, and nephrotoxicity [37] induced by malathion. On the contrary, vitamin C and vitamin E did not ameliorate nephrotoxicity induced by malathion [38]. The impact of estradiol against nephrotoxicity induced by malathion has been inadequately described. In addition, recent studies that investigated the protective effect of estradiol against nephrotoxicity induced by other drugs produced contrasting results. Estradiol promoted nephrotoxicity induced by cisplatin (CP) in rats [39]. In another in vivo study, estradiol failed to inhibit CP-induced nephrotoxicity [40]. On the contrary, estradiol demonstrated a protective effect against a nephrotoxicity model induced by gentamicin in rats [41] and against a nephrotoxicity model induced by methotrexate in human renal epithelium cells [42]. These protective effects resulted from the potent antioxidant properties of this hormone. Estradiol contains phenolic hydroxyl groups that prevent cell damage by donating hydrogen atoms to free radicals, particularly lipid peroxyl radicals, thereby suppressing their activity, which is the primary cause of cell membrane damage [19]. In addition, estradiol stimulates the activity of natural cellular antioxidant enzymes [43]. Ascorbic acid acts as a powerful antioxidant through three mechanisms: removal of free radicals, formation of antioxidant system enzymes, and the repair of damaged tissues following oxidation [44]. Alhilal and Salem [19] found that ascorbic acid can rebalance the oxidant/antioxidant system and restore the antioxidant properties of oxidized tocopherol. Ascorbic acid showed stronger effects compared to estradiol, which is consistent with the results of our previous study [19].

In the present study, the inhibition of AChE was the primary mechanism of the toxic effects of malathion by increasing acetylcholine accumulation at cholinergic synapses and neuromuscular junctions. Accordingly, our study highlights the molecular docking analysis of estradiol and ascorbic acid against the selected protein (PDB ID: 2YMX). The importance of our molecular docking study may be summarized by the following points: First, the results support the superiority of ascorbic acid over estradiol. Second, molecular docking provides a hypothesis for this superiority, evidenced by the greater ability of ascorbic acid to hinder the target enzyme (PDB ID: 2YMX), which inhibits AChE by targeting the backdoor region. The reactivation of inhibited AChE represents an important strategy for reducing acetylcholine accumulation at cholinergic synapses and neuromuscular junctions. Furthermore, molecular docking revealed that ascorbic acid exhibits a higher capacity for electron donation compared with estradiol. This hypothesis reinforces the ability of ascorbic acid to scavenge free radicals and mitigate oxidative stress, which is an important factor in the toxic effects of malathion.

Determining the relationship between kidneys and estrogen will enhance our understanding of these findings. Estrogen receptors, and particularly estrogen receptor α, are highly expressed in the kidney, which is considered a nonreproductive estrogenic organ [45,46]. Many important physiological functions occur in the kidney that are mediated by estrogen and its receptors, such as mitochondrial homeostasis, tissue repair, and regeneration [47]. Abd El-Lateef et al. [46] reported that estrogen depletion following ovariectomy in rats and the suppression of estrogen receptors in the kidney were associated with the development of gentamicin-induced acute kidney injury, which resulted from increased megalin expression in the kidney. The injury was ameliorated by treatment with estrogen (17β-estradiol) and its receptors.

Estradiol was used in this study because it is commonly used to treat disorders of the female reproductive system. Treating menopause and its side effects is one of the most important therapeutic effects of estradiol; however, it cannot be used in men because it affects the natural male hormonal system. Consequently, the results of estradiol in our study may only be applied to women. Our findings may have clinical significance for some women after more comprehensive studies are conducted. For example, postmenopausal women receiving estradiol therapy may have a reduced tendency to develop nephrotoxicity when exposed to malathion. Moreover, women who use estradiol to treat other reproductive disorders can benefit from its potential antinephrotoxic effects following exposure to malathion.

Ascorbic acid is also effective with no side effects. It is affordable and widely available. Therefore, it may be used as a preventive agent to reduce kidney damage in patients who are exposed to high concentrations of malathion.

This study has some specific limitations as follows: We compared the protective roles of ascorbic acid and estradiol against kidney damage induced by malathion through histopathology and molecular docking analysis; however, some biomarkers such as oxidative stress indicators were not evaluated, which was a limitation of the study. Similarly, while we used estradiol and ascorbic acid in different groups, adding one more experimental group describing the combined administration of estradiol and ascorbic acid would have expanded the conclusions drawn from this investigation. Further studies of these substances and comprehensive in vivo studies are needed to provide further insight into the precise biochemical mechanisms supporting the superiority of ascorbic acid over estradiol, in particular by targeting the specific signaling pathways associated with inflammation and oxidative stress by ascorbic acid and estradiol.

In conclusion, estradiol and ascorbic acid exhibit protective effects against malathion-induced kidney injury, with a more pronounced protective role observed for ascorbic acid. Ascorbic acid may be used as a preventive agent to reduce kidney damage in individuals who are exposed to malathion. Postmenopausal women receiving estradiol therapy may have a reduced tendency to develop nephrotoxicity when exposed to malathion, although additional in vivo studies are necessary to support these findings and their clinical relevance. Molecular docking results also revealed the superiority of ascorbic acid over estradiol (ascorbic acid displayed the highest docking score of −6.20 kcal/mol) and provided insight into the mechanism by which ascorbic acid suppresses nephrotoxicity resulting from malathion exposure.

## Figures and Tables

**Table:** Docking score (kcal mol^−1^), number of H bonds formed between designed compounds and 2YMX receptor, and RMSD compared to native cocrystallized ligand GOL.

Entry	Docking score (kcal/mol)	Hydrogen bonds	Donor atom	Acceptor atom	RMSD, kcal mol^−1^ Å^−1^
Estradiol	−5.12	1 (Asp 112)	O	–	1.89
Ascorbic acid	−6.44	2 (Asp 165) 1 (Tyr 173)	O	–	1.17
CCL	−4.80	1 (Asp 165) 1 (Lys 142)	O	O	1.31
